# SEQUENTIAL ANALYSIS OF DEMENTIA FAMILY DYADIC COMMUNICATION: CARE RECIPIENT ENGAGING COMMUNICATION

**DOI:** 10.1093/geroni/igae098.0445

**Published:** 2024-12-31

**Authors:** Sohyun Kim, Will Chen, Shan Sun-Mitchell

**Affiliations:** The University of Texas Arlington, Arlington, Texas, United States; The University of Texas Arlington, Arlington, Texas, United States; The University of Texas Arlington, Arlington, Texas, United States

## Abstract

Persons living with dementia (care-recipient) engaging communication is responsive and collaborative communication. Examining dementia family dyadic communication is useful for prioritizing caregiver response to care-recipient communication. This study examined temporal relationships between antecedent care-recipient engaging communication and subsequent family caregiver communication (facilitative, disabling, and neutral) during 75 in-home care video observations. A secondary analysis of 5- to 60-second timed-window analysis with 5-second intervals was conducted. 95% confidence intervals, p-values, and Yule’s Q statistics were used. The results showed all types of caregiver communication were less likely to occur within all time widows after care-recipient engaging verbal communication (OR = 0.19– 0.43, p < 0.001, Yule’s Q = -0.398 – -0.685). Caregiver facilitative verbal, disabling verbal and nonverbal, and neutral verbal communication were less likely to occur within all time widows after care-recipient engaging nonverbal communication (OR = 0.37 – 0.83, p < 0.001 – p = 0.017, Yule’s Q = -0.093 – -0.459). Caregiver facilitative nonverbal communication was less likely to occur until 35-second after care-recipient engaging nonverbal communication (OR = 0.67 – 0.81, p < 0.001 – p = 0.001 – 0.020, Yule’s Q = -0.099 – -0.198). Care-recipient engaging communication was significantly associated with a decreased likelihood of subsequent caregiver communication, with a small to large probability of non-occurrence. Caregivers failed to respond to care-recipient communication, regardless of its nature. This emphasizes the urgent need for healthcare providers and researchers to prioritize caregiver education to promote a more supportive and engaging environment for persons living with dementia.

